# E2 regulates MMP-13 via targeting miR-140 in IL-1β-induced extracellular matrix degradation in human chondrocytes

**DOI:** 10.1186/s13075-016-0997-y

**Published:** 2016-05-10

**Authors:** Yujie Liang, Li Duan, Jianyi Xiong, Weiming Zhu, Qisong Liu, Daming Wang, Wei Liu, Zigang Li, Daping Wang

**Affiliations:** School of Chemical Biology and Biotechnology, Peking University Shenzhen Graduate School, Shenzhen, 518055 Guangdong Province China; Departments of Chemistry, The Chinese University of Hong Kong, Shatin, Hong Kong, SAR China; Shenzhen Key Laboratory of Tissue Engineering, Shenzhen Second People’s Hospital (The First Hospital Affiliated to Shenzhen University), Shenzhen, 518035 Guangdong Province China; Department of Orthopedics, Shenzhen Second People’s Hospital (The First Hospital Affiliated to Shenzhen University), Shenzhen, 518035 Guangdong Province China

**Keywords:** Menopausal arthritis, Estrogen, miR-140, MMP-13

## Abstract

**Background:**

Estrogen deficiency is closely related to the development of menopausal arthritis. Estrogen replacement therapy (ERT) shows a protective effect against the osteoarthritis. However, the underlying mechanism of this protective effect is unknown. This study aimed to determine the role of miR-140 in the estrogen-dependent regulation of MMP-13 in human chondrocytes.

**Methods:**

Primary human articular chondrocytes were obtained from female OA patients undergoing knee replacement surgery. Normal articular chondrocytes were isolated from the knee joints of female donors after trauma and treated with interleukin-1 beta (IL-1β). Gene expression levels of miR-140, MMP-13, and ADAMTS-5 were detected by quantitative real-time PCR (qRT-PCR). miR-140 levels were upregulated or downregulated by transfecting cells with a miRNA mimic and inhibitor, respectively, prior to treatment with IL-1β. MMP-13 expression was then evaluated by Western blotting and immunofluorescence. Luciferase reporter assays were performed to verify the interaction between miR-140 and ER.

**Results:**

17-β-estradiol (E2) suppressed MMP-13 expression in human articular chondrocytes. miR-140 expression was upregulated after estrogen treatment. Knockdown of miR-140 expression abolished the inhibitory effect of estrogen on MMP-13. In addition, the estrogen/ER/miR-140 pathway showed an inhibitory effect on IL-1β-induced cartilage matrix degradation.

**Conclusions:**

This study suggests that estrogen acts via ER and miR-140 to inhibit the catabolic activity of proteases within the chondrocyte extracellular matrix. These findings provide new insight into the mechanism of menopausal arthritis and indicate that the ER/miR-140 signaling pathway may be a potential target for therapeutic interventions for menopausal arthritis.

**Electronic supplementary material:**

The online version of this article (doi:10.1186/s13075-016-0997-y) contains supplementary material, which is available to authorized users.

## Background

Osteoarthritis (OA) is the most common articular pathology and is the leading cause of mobility-associated disability, which is characterized by pain, tenderness, limited movement, crepitus, and a variable degree of inflammation without systemic effects [1]. The pathogenesis of OA is complex and involves the interaction of multiple factors. Among these factors, sex hormones play critical roles in OA pathogenesis in women over 50 years old or after menopause. The ‘arthritis of menopause’ phenomenon, where OA incidence rises steeply with age after menopause [2–4], was described as early as 1926. The prevalence of OA increases in women with menopausal transition and is thought to be due to a reduction of estrogen levels [5, 6]. However, the effect of estrogen replacement in OA patients and the correlation between estrogen and OA are unclear [7–9]. Several studies report that estrogen exerts a chondroprotective effect, while others present evidence of a chondrodestructive effect or no effect on cartilage. OA is characterized by degradation of extracellular matrix (ECM) macromolecules by proteolytic enzymes produced by activated chondrocytes, including matrix metallopeptidase 13 (MMP-13) [10–12].

Both estrogen receptor (ER) and MMP-13 are expressed in joint tissue and chondrocytes, indicating that chondrocytes can respond to estrogen treatment [13, 14]. However, studies on the effect of ovariectomy (OVX) and estrogen treatment on cartilage in animal models of OA were inconclusive [15]. Gene expression of some extracellular matrix molecules was inhibited by estrogen. Physiological levels of estrogen decreased MMP-1 expression and increased proteoglycan synthesis in chondrocytes [15, 16]. MMP-13 microRNA (mRNA) levels were significantly suppressed by 17-β-estradiol (E2) in articular chondrocytes of female patients [17]. Further investigation is needed to understand the mechanisms underlying the effect of estrogen on matrix proteases and to develop effective therapeutic targets for OA.

miRNAs are a class of abundant, small, highly conserved noncoding RNA (approximately 22 nucleotides) found in higher eukaryotes. They act as multifunctional mediators of biological processes by partially base-pairing with mRNA sequences of target genes in the 3′-untranslated region (UTR) [18, 19], thus contributing to posttranscriptional regulation of gene expression. Accumulating data have demonstrated that miRNAs are critical for pathogenesis developmental and cellular processes, including organogenesis and differentiation [20–22]. Recently, miRNA families have been shown to target genes expressed in cartilage and have therefore been studied to elucidate their involvement in cartilage tissue development, homeostasis, and degradation [23–26]. A select number of miRNAs play key roles in the development of OA [27, 28]. For example, miR-140 directly targets the gene that encodes a disintegrin and metalloproteinase with thrombospondin motifs 5(ADAMTS-5), an aggrecanase that cleaves aggrecan in cartilage. miR-140 is also a negative regulator of MMP-13, and miR-140 expression is downregulated in OA [29–32]. However, the mechanism that underlies miR-140-mediated regulation upstream of its effects on ADAMTS-5 and MMP-13 is currently unclear. The transcription factors involved in miR-140 expression in OA pathogenesis are also unknown.

ER is a ligand-activated transcription factor that regulates target genes at both transcriptional and posttranscriptional levels. For example, following estrogenic activation, ER directly regulates the transcripts of some target genes via DNA binding [33]. In contrast to hormonally regulated transcription, which has been extensively described at the molecular level, regulation of miRNA by estrogenic activation of ER has not been widely studied [34].

In this study, we sought to determine whether estrogen regulates MMP-13 expression and interleukin-1 beta (IL-1β)-induced catabolic responses in human chondrocytes. Our results demonstrate that miR-140 acts as an intermediary transcriptional factor to suppress MMP-13 and IL-1β responses in human chondrocytes. Thus, miR-140 may be a regulator of cartilage homeostasis in OA. Estrogen-bound estrogen receptor alpha (ERα) was shown to initiate the transcription of miR-140 by associating with its ER response element (ERE) element.

## Methods

### Primary chondrocytes culture

The donation of cartilage was approved by the ethics committee at the Medical Faculty at Shenzhen Second People’s Hospital. Informed consent had been obtained from cartilage donors. OA cartilage samples was obtained from nine female patients (mean age > 53 years) who underwent joint replacement or joint surgery in the department of Orthopedics, Shenzhen Second People’s Hospital. Articular cartilage tissues were cut into small fragments (1–2 mm^3^), and followed by digestion with 1 mg/mL collagenase (Sigma-Aldrich, St. Louis, MO, USA) for 16 h at 37 °C with shaking. After dissociation, the triturate suspension was filtered through a 100-mm nylon cell strainer (Falcon, BD, Oxford, UK) to remove matrix debris, and cells were collected by centrifugation at 800 × g for 10 minutes. Chondrocytes were then resuspended in Dulbecco’s modified Eagle’s medium (DMEM)/F-12 medium (Gibco, Invitrogen, Carlsbad, CA, USA), supplemented with 10 % fetal bovine serum (FBS), 100 IU/ml penicillin, and 100 μg/ml streptomycin (Gibco). Primary cultures of human chondrocytes and treatment passage 0 cells were subcultured after 5–7 days of isolation and either used directly (P1 cells) or passaged once more (P2 cells) before experiments. Chondrocytes were serum starved overnight and then treated with 5 ng/mL recombinant human IL-1β (PeproTech, London, UK) or E2 for the indicated periods of time. Chondrocytes were placed in monolayer culture in 12-well plates (for RNA) or 6-well plates (for protein). Transfection experiments were performed 1 day after seeding.

### Treatment with IL-1β and estrogen

For OA chondrocytes from female patients, primary OA chondrocytes at 80 % confluence were used for the experiments. These cells were incubated with indicated concentration of E2 (Sigma E-8875, Sigma-Aldrich). Stock solutions of 10^−1^M E2 in absolute ethanol were prepared and diluted stepwise. Controls were incubated with 0.1 % ethanol instead of the hormones. For normal chondrocytes from trauma patients (*n* = 3), primary cultured human chondrocytes were serum starved overnight and then treated with recombinant human IL-1β (5 ng/mL; PeproTech) for the indicated periods of time for incubations with estradiol.

### RNA extraction, reverse transcription, and quantitative real-time (RT)-PCR

Total RNA from human articular chondrocytes was extracted with TRIzol reagent (Invitrogen) according to the manufacturer’s instructions. Then 1 μg total RNA was reverse-transcribed with a specific stem-loop primer for miRNA and with a random primer for messenger RNA (mRNA) using reverse transcription kits (Takara Biotechnology, Dalian, China), respectively. The mRNA expression was analyzed using qRT-PCR with Sybr Green premix (Takara Biotechnology). For the reverse transcription step, a miRNA-specific stem-loop primer (= looped primer) was used (5 pmol in 20 μl reaction volume) to generate cDNA. Glyceraldehyde 3-phosphate dehydrogenase (GAPDH) and small nuclear RNA U6 were used as internal controls for complementary DNA (cDNA) and miRNA, respectively [35]. Primer sequences used for qRT-PCR are listed in Table 1.

**Table 1:** Primers used for stem-loop reverse transcription or polymerase chain reaction of microRNAs and messenger RNA

Gene name		Primer sequences (5′-3′)
	Sense	GTAGGCACTGCAGATGAGAGAGAGAGAGCGCTGT
	Antisense	ATCGACAAGCTTCATGCTGCCTTCAGATGAGA
miR-140-5p	Stem-loop RT primer	GTCGTATCCAGTGCAGGGTCCGAGGTATTCGCACTGGATACGACCTACCAT
	Sense	CGCGCCAGTGGTTTTACCCT
	Antisense	CCAGTGCAGGGTCCGAGGTA
*U6*	Stem-loop RT primer	GTCGTATCCAGTGCAGGGTCCGAGGTATTCGCACTGGATACGACACGATT
	Sense	CCTGCGCAAGGATGAC
	Antisense	GTGCAGGGTCCGAGGT
MMP-13	Sense	TGATGACATCAAGAAGGTGGTGAAG
	Antisense	TCCTTGGAGGCCATGTGGGCCAT
GAPDH	Sense	GATCATCAGCAATGCCTCCT
	Antisense	TGTGGTCATGAGTCCTTCCA

*GAPDH* glyceraldehyde 3-phosphate dehydrogenase, *MMP-13* metalloproteinase 13

### Immunofluorescence microscopy

Immunostaining to detect expression of MMP-13 in chondrocytes was performed using standard protocols. Chondrocytes were cultured on coverslips under different treatment. Briefly, cells were fixed with 4.0 % paraformaldehyde (Sigma-Aldrich) and permeabilized with 0.1 % Triton X100 for 10 min (Sigma-Aldrich) in Dulbecco’s phosphate-buffered saline (Invitrogen). Nonspecific binding was blocked with 10 % goat serum, and cells were incubated overnight with MMP13 primary antibody at a dilution of 1:200. After washing, the cells were incubated with anti-mouse Alexa Fluor 594 (Invitrogen) secondary antibodies (1:300) for 1 h at room temperature. Coverslips were mounted on slides and observed using a fluorescent microscope. We used DAPI (Sigma-Aldrich; 1 μg/ml) for nuclear staining.

### Western blotting

Cells were lysed with lysis buffer (50 mM Tris-HCl, pH 7.4, 150 mM NaCl, 1 % NP- 40, and 0.1 % sodium dodecyl sulfate) supplemented with protease inhibitor cocktail set I (Biotool, Jupiter, FL, USA) and phenylmethanesulfonyl fluoride (PMSF, Sigma-Aldrich), and the concentration was measured using the BCA protein assay kit (Pierce, Rockford, IL, USA) using bovine serum albumin as the standard. Proteins were fractionated by SDS polyacrylamide gel electrophoresis and transferred onto a PVDF membrane. The membrane was blocked with 5 % nonfat dry milk in Tris-buffered saline containing 0.1 % Tween 20. MMP13 protein was detected by Western blotting using a polyclonal anti-MMP13 antibody (abcam, ab39012, Abcam, Cambridge, MA, USA) and ECL reagent (EMD Millipore, Billerica, MA, USA) according to the manufacturer’s instructions.

### miRNA and RNAi transfection

Chondrocytes were plated in 6-well plates at 2 × 10^5^ per well for transfection. Twenty-four hours after plating, 100 nmol of has-miR-140-5p mimic or 100 nM scrambled 22 nt nucleotides (miR-Scr, with no homology to mammal genome) or 150 nM inhibitors (designed and synthesized by RiboBio, Guangzhou, China) were transfected to the cells with Lipofectamine RNAiMAX (Invitrogen) following the manufacturer’s protocol. miR-Scr was transfected as negative controls. The transfection efficiency was quantified by miR-140-5p qRT-PCR. After 12 hours of incubation, the cells were used for the following experiments.

RNA interference-mediated ERα gene silencing. ERα gene-specific small interfering RNA (siRNA) (EHU141651) were purchased from Sigma-Aldrich. Transfection was performed at a final concentration of 20 nmol/l using Lipofectamine RNAiMAX (Invitrogen). The expression level of ERα in cell transfected with ERα RNAi decreased by 36.4 % respectively when compared with the cells without transfection.

### Luciferase assay

All reporter plasmids for transfection were prepared using the Qiagen plasmid purification kit (Qiagen, Hilden, Germany). To confirm the activation effect of miR-140 promoter activity, SW1353 human chondrosarcoma cells were transiently transfected using Lipofectamine 2000 (Invitrogen) according to the manufacturer’s instructions. Twelve hours after transfection, the cells were serum starved for 12 hours followed by 4 hours treatment with or without E2. Then cell lysates were extracted, and Firefly luciferase activities were measured using the Luciferase Reporter Assay System (Promega, Madison, WI, USA) according to the manufacturer’s protocol. Firefly luciferase activity was normalized to protein concentrations. Each experiment was performed three times in triplicate.

### Statistics

All experiments were repeated three times with independent cultures and similar results were obtained. Real-time PCR data were given as a threshold cycle (Ct). Fold changes in gene expression were calculated as 2^-ΔΔCt^. Statistical significance was evaluated using a two-tailed Student’s *t* test. All data are shown as mean + SE. *P* < 0.05 was considered statistically significant.

## Results

### ER induction upregulates miR-140 expression

**Fig. 1:**
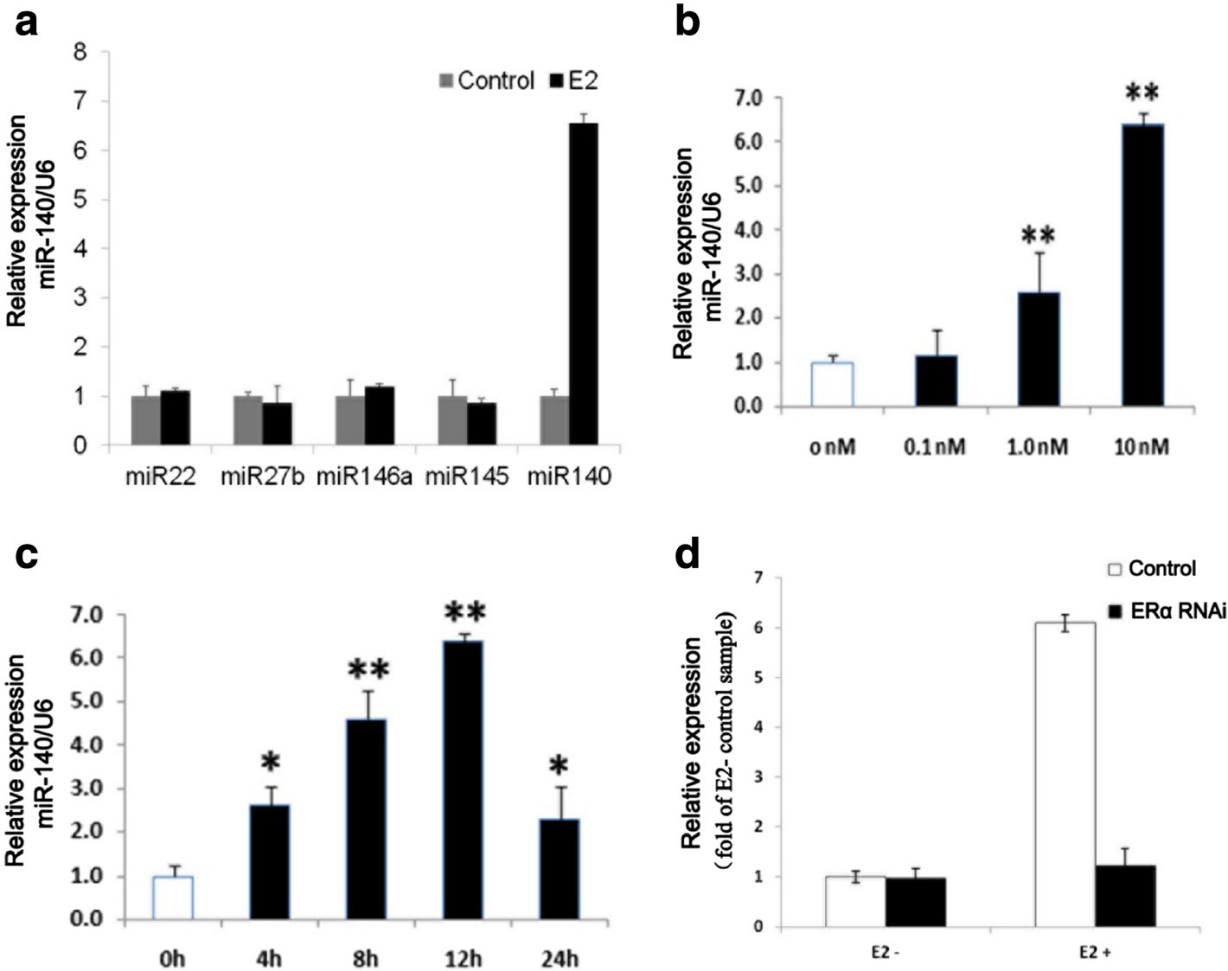
E2 induced miR-140 expression in time and concentration dependent. **a** Influence of E2 (10 nM) on OA-related miRNA expression in primary human articular chondrocytes from female OA patients. **b** Relative expression of miR-140 in female OA chondrocytes stimulate with different concentration of E2 for 12 hr. **c** Kinetics of expression of miR-140 expression treated with E2 (10 nM) for 4, 8, 12 and 24 hr in OA chondrocytes from female patients. **d** E2 induction of miR-140 expression was significantly repressed by estrogen receptor alpha RNAi. Data are represented as mean ± SEM, statistically different from control (^*^ *P* < 0.05, ^**^ *P* < 0.001). *E2* 17-β-estradiol, *ERα* estrogen receptor alpha

To determine whether ER regulates the expression of miRNAs, E2 was added to chondrocytes and miRNA expression was evaluated by quantitative real-time PCR (qRT-PCR). Since miR22, miR27b, miR-145, miR-146a, and miR-140 have been linked to the pathophysiology of OA [36], we first evaluated the expression of these miRNA following E2 stimulation. There was no difference in the expression levels of miR22, miR27b, miR-145, or miR-146a before or after E2 treatment (Fig. 1a). However, the expression of miR-140 was upregulated in a concentration-dependent manner (Fig. 1b) miR-140 expression in primary chondrocytes was rapidly induced (within 4 hours) by E2 treatment, and expression levels gradually increased over a 12-hour period (Fig. 1c). Thus, these data present new evidence that miR-140 is an early ER-regulated gene. E2 induction of miR-140 expression was significantly repressed by estrogen receptor alpha RNAi (Fig. 1d).

### E2 downregulates the expression of MMP-13 and ADAMTS-5 in OA chondrocytes

**Fig. 2:**
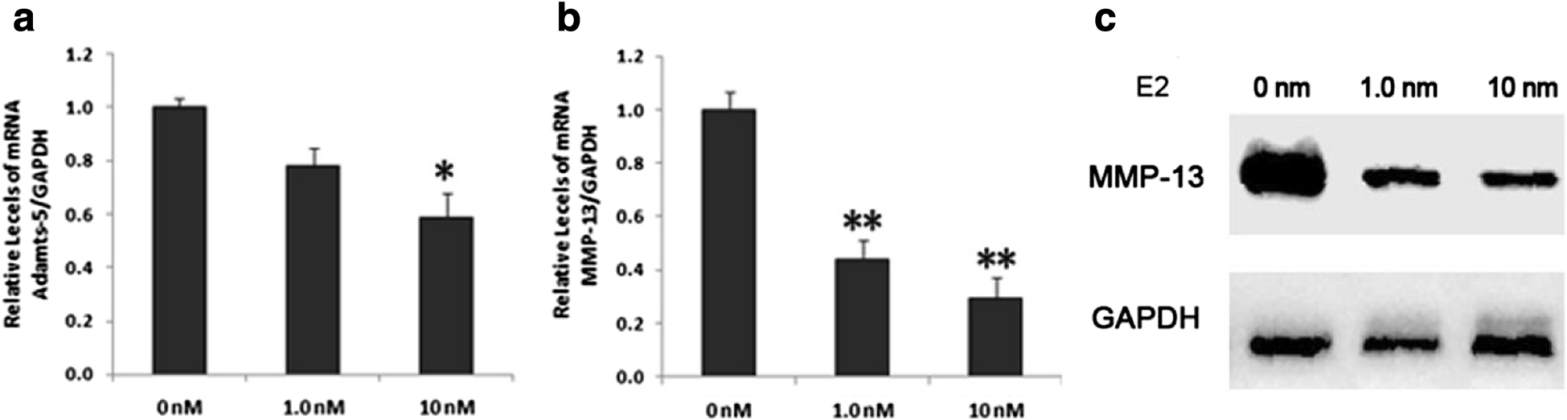
E2 suppresses MMP-13 expression in female OA chondrocytes. **a** Relative expression levels of Adamts-5 mRNA 24 hr after E2 treatment in primary OA chondrocytes from female patients. **b** Relative expression levels of MMP-13 mRNA 24 hr after E2 treatment in primary OA chondrocytes from female patients. **c** Western blot analyses of MMP-13 protein expression in OA chondrocytes from female patients were treated with or without E2. Data are represented as mean ± SEM. ^*^ *P* < 0.05, ^**^ *P* < 0.001. *E2* 17-β-estradiol, *GAPDH* glyceraldehyde 3-phosphate dehydrogenase, *MMP-13* metalloproteinase 13, *mRNA* messenger RNA

Previous studies reported that miR-140 expression was lower in OA cartilage than in normal cartilage [31]. Consistent with these previous reports our study showed that the expression of MMP-13 was increased in OA cartilage compared to normal cartilage (Additional file 1: Figure S1). We studied the effect of E2 on MMP-13 expression levels in the chondrocytes of OA patients (age range: 50–70 years, female). Chondrocytes were treated with different concentrations of E2, and MMP-13 gene expression was determined by qRT-PCR, with MMP-13 levels normalized to GAPDH levels. E2 significantly inhibited MMP-13 expression levels in OA chondrocytes in a dose-dependent manner (Fig. 2a and b).

### IL-1β-induced expression of MMP-13 and miR-140 is blocked by E2

**Fig. 3:**
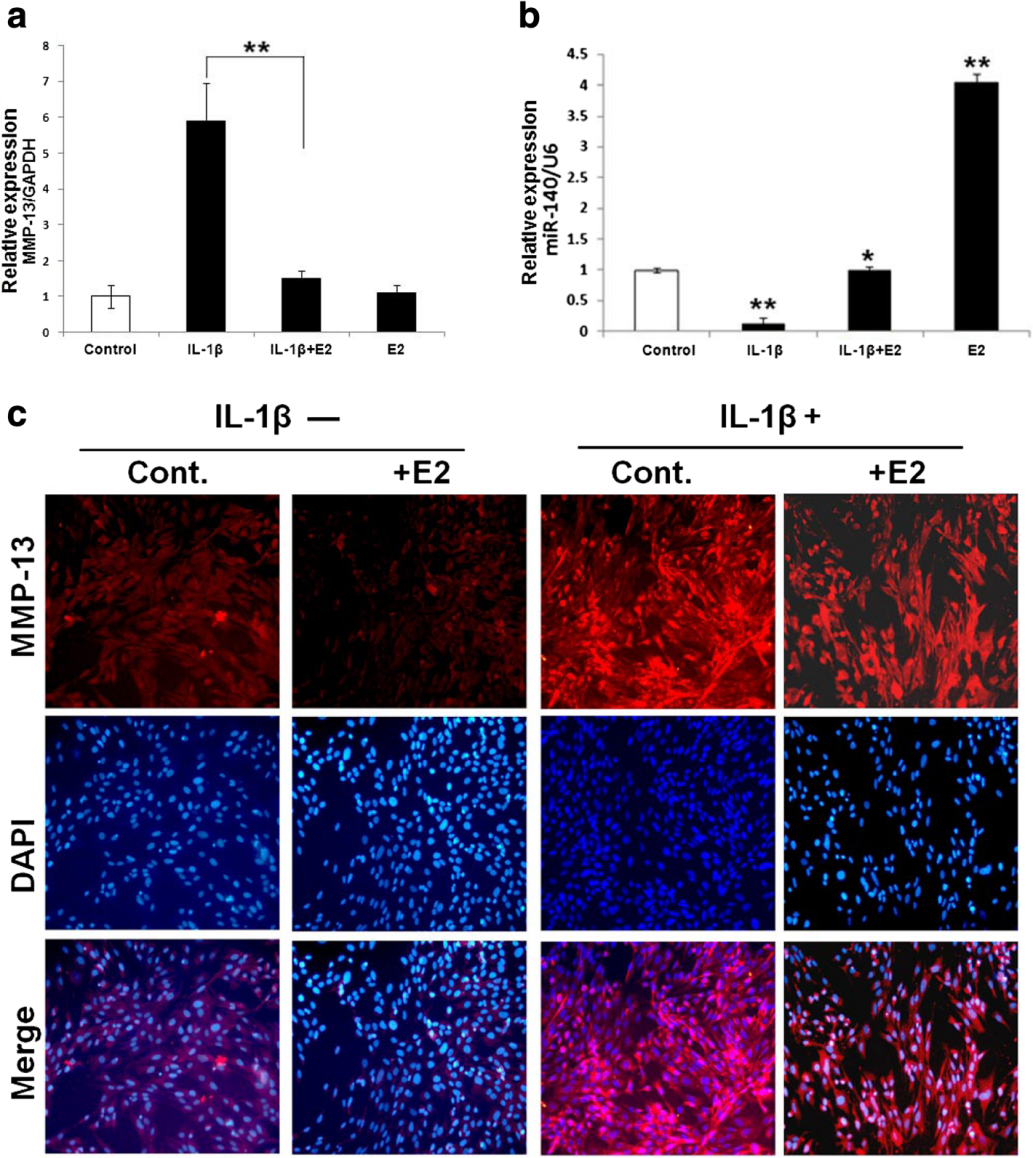
Effect of E2 on MMP-13 and miR-140 expression in IL-1β-stimulated chondrocytes. **a** Relative expression of MMP-13 in normal chondrocytes stimulated with IL-1β (5 ng/ml) for 4 hr, then treated with 10 nM E2 for 24 hr. **b** Relative expression of miR-140 in normal chondrocytes pretreated with IL-1β (5 ng/ml) for 4 hr, then treated with 10 nM E2 for 24 hr. **c** Immunofluorescence staining of MMP-13 expression in normal chondrocytes pretreated and untreated with IL-1β (5 ng/ml) for 4 hr, then treated with 10 nM E2 for 24 hr. Data are represented as mean ± SEM. ^*^ *P* < 0.05, ^**^ *P* < 0.001. *Cont* control, *E2* 17-β-estradiol, *GAPDH* glyceraldehyde 3-phosphate dehydrogenase, *IL-1β* interleukin-1 beta, *MMP-13* metalloproteinase 13

MMP-13 plays an important role in the degradation of cartilage, especially in IL-1β-stimulated human OA chondrocytes. Normal chondrocytes treated with IL-1β displayed degenerative characteristics. To further analyze the effect of E2 on IL-1β-induced extracellular matrix (ECM) degradation, chondrocytes were pretreated with IL-1β to induce MMP-13 expression. The effect of E2 on IL-1β-induced MMP-13 expression at the gene and protein levels was evaluated by qRT-PCR and immunofluorescence staining, respectively. These results showed that IL-1β (5 ng/ml) significantly induced MMP-13 expression at both the mRNA and protein levels. After treatment with E2 (10 nM), gene and protein expression of MMP-13 was significantly reduced (Fig. 3a  and c). Subsequently, the expression of miR-140 in IL-1β-stimulated human chondrocytes was quantified. As expected, miR-140 expression was significantly decreased in chondrocytes following IL-1β stimulation. However, this downregulation of miR-140 expression was blocked by E2 (Fig. 3b).

### E2 modulates MMP-13 transcript expression via miR-140 in human chondrocytes

**Fig. 4:**
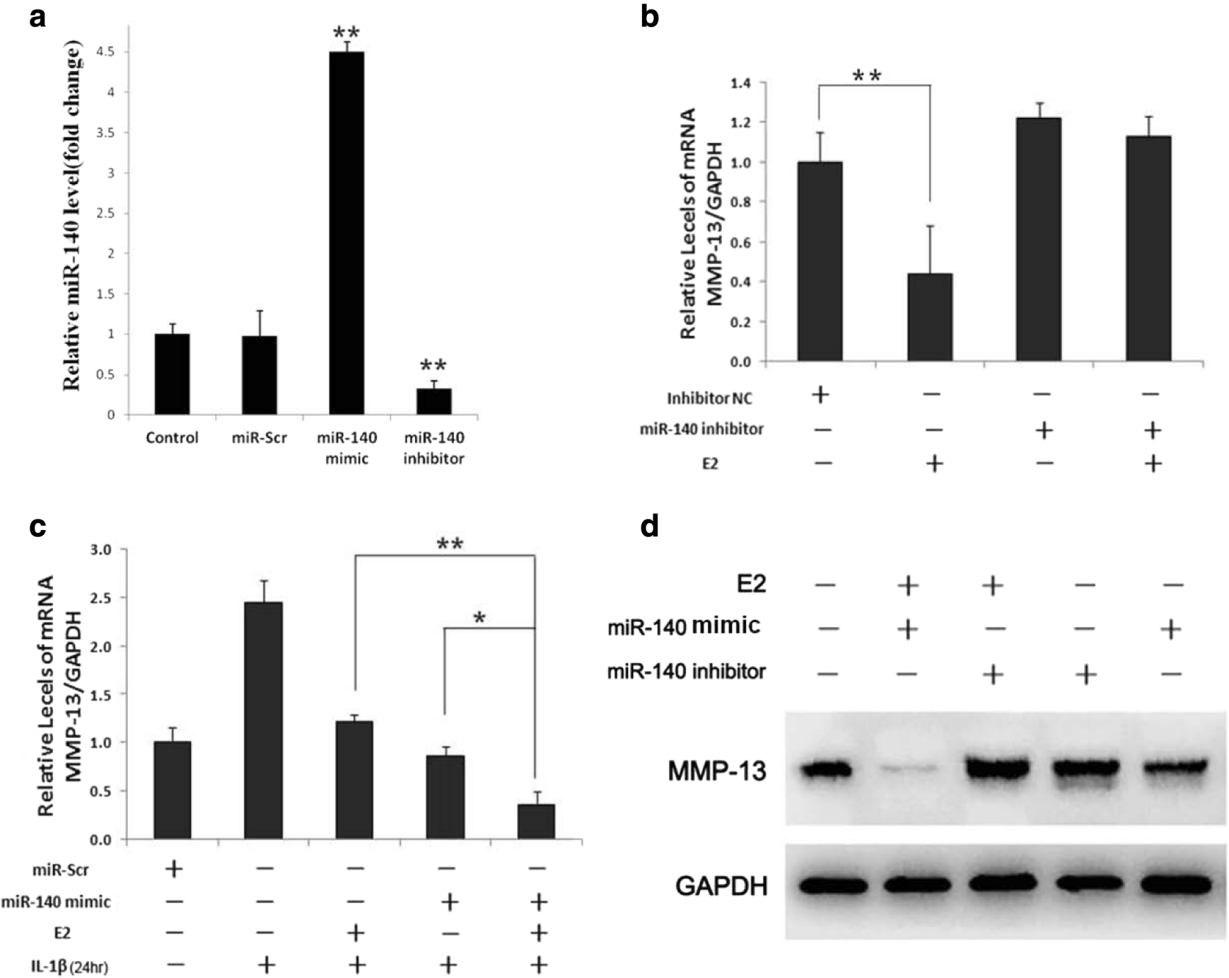
E2 acts via the ER/miR-140 pathway to suppress IL-1β-mediated catabolic responses in chondrocytes. **a** Transfection efficiency of miR-140 inhibitor and miR-140 mimic in chondrocytes. Chondrocytes (2.5 × 10^4^ cells per well in a 24-well plate) were planted and transfected with miR-140 inhibitor, miR-140 mimic, and their negative controls (miR-Scr) up to 24 hr. **b** Relative MMP-13 mRNA in normal chondrocytes transfected with miR-140 inhibitor then stimulated with IL-1β (5 ng/ml) for 4 hr, then treated with 10 nM E2 for 24 hr. Inhibitor NC was set to one, as control. **c** Relative expression of MMP-13 in normal chondrocytes transfected with miR-140 mimic and pretreated with IL-1β (5 ng/ml) for 4 hr, then treated with 10 nM E2 for 24 hr. miR-scramble was set to one, as control. **d** Western blot of MMP-13 expression in normal chondrocytes transfected with miR-140 inhibitor or miR-140 mimic pretreated and untreated with IL-1β (5 ng/ml) for 4 hr, then treated with10 nM E2 for 24 hr. GAPDH was used as internal control. Data are represented as mean ± SEM. ^*^ *P* < 0.05, ^**^ *P* < 0.001. *E2* 17-β-estradiol, *GAPDH* glyceraldehyde 3-phosphate dehydrogenase, *IL-1β* interleukin-1 beta, *MMP-13* metalloproteinase 13

Our data demonstrated that E2 can suppress MMP-13 expression and upregulate miR-140 expression. We hypothesized that the inhibiting effect of E2 on MMP-13 may be mediated by miR-140. To examine the role of miR-140 in the downregulation of MMP-13 by E2 and to determine whether modulation of miR-140 can control the pathogenesis of OA in vitro, chondrocytes were transfected either with a miR-140 mimic or a miR-140 inhibitor. After transfection, cells were stimulated with IL-1β for the indicated times. Pre-transfection of IL-1β-induced chondrocytes with the miR-140 inhibitor abolished the E2-mediated reduction of endogenous levels of MMP-13 transcripts and proteins (Fig. 4b and d). Interestingly, miR-140 overexpression by transfection with the miR-140 mimic significantly reduced the expression of MMP-13 in E2-treated IL-1β-stimulated human chondrocytes (Fig. 4c and d) Thus, our results suggest that miR-140 is a crucial regulator of E2-mediated cartilage homeostasis, where miR-140 enables E2 to suppress IL-1β-induced MMP-13 production. Taken together, these data strongly indicate that E2 regulates MMP-13 gene expression in human chondrocytes via the miR-140 regulatory pathway. Thus, miR-140 expression was found to be necessary for the significant inhibitory effect of E2 on IL-1β-induced MMP-13 expression

### E2 promotes miR-140 expression by activating ERE

**Fig. 5:**
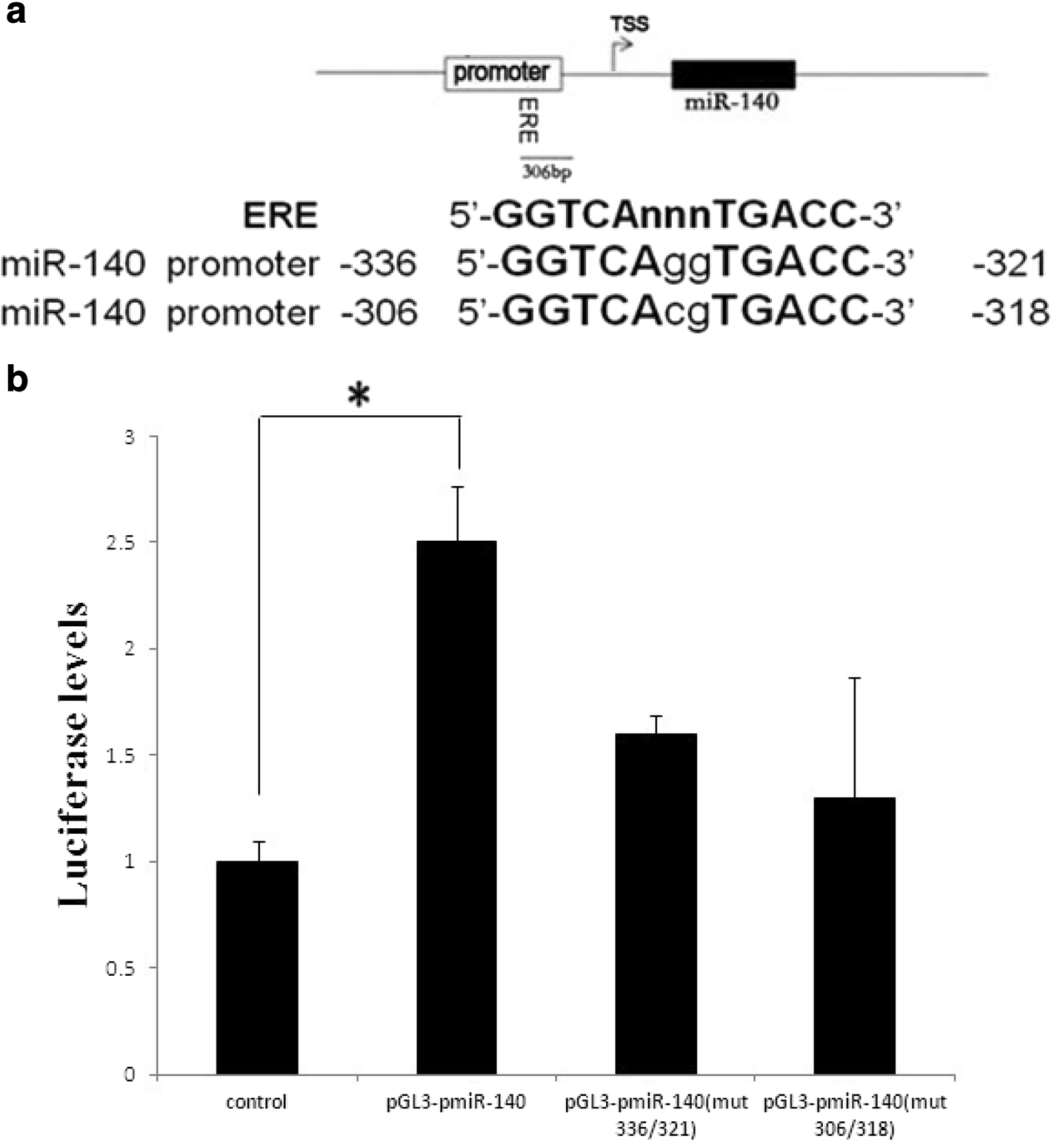
E2 stimulation activates human miR-140 promoter activity and is mediated by the −336/-321- and −306/-318- bp sequences. **a** −495 proximal region sequence upstream of the miR-140 star sequence contains two classical estrogen response elements with the consensus sequence 5′-GGTCAnnnTGACC-3′. **b** Luciferase activity in SW1353 cells transfected with miR-140 promoter wide-type (pGL3.-pmiR-140) and two miR-140 promoter mutants (mut −336/-321 and mut −306/-318), 12 hr after transfection, the cells were serum starved for 12 hr followed by 4-hr treatment with E2 (10 nM). The date are expressed as mean and SEM of three independent experiment. ^*^Statistically different from control (*P* < 0.05). *ERE* estrogen response element, *TSS* transcription start site

In order to determine whether the upregulation of miR-140 is transcriptionally dependent on E2, we analyzed the promoter region of miR-140 and found two putative ERE binding sites for ER in the region upstream of miR-140, suggesting that ER may modulate miR-140 expression directly (Fig. 5a). The regulation of miR-140 by ER was further investigated using a reporter plasmid containing the Firefly luciferase gene under control of the −495 proximal region sequence upstream of the start codon for miR-140. E2 stimulation induced activation of ER, which then positively regulated luciferase activity (Fig. 5b). We then performed a luciferase assay to confirm that the two putative ER binding sites do indeed respond to ER. Compared to the wild-type miR-140 promoter, two miR-140 promoter mutants (mut −336/-321 and mut −306/-318) exhibited low response to E2. This indicated that the 495 bp region upstream from miR-140 is required for its regulation by E2. Altogether, these results demonstrated that the E2 ligand activates the transcription factor ER, which then binds to specific ERE sequences in the promoter region of miR-140 and positively enhances its transcription.

## Discussion

The primary pathogenic event in OA is aberrant remodeling of the cartilage ECM. Estrogen replacement therapy has been employed to treatment of knee and hip osteoarthritis. However, the underlying molecular mechanisms by which estrogen regulates cartilage ECM remain unclear. MMP-13 is known to play a crucial role in IL-1β-induced cartilage matrix degradation. Moreover, miR-140 inhibits MMP-13 expression in articular chondrocytes [37]. In the present study, we confirmed that E2 has the capacity to downregulate MMP-13 transcript and protein levels in OA-diseased chondrocytes from female patients. We further demonstrated that E2 treatment suppresses MMP-13 expression induced by IL-1β in normal chondrocytes, indicating that E2 may reduce cartilage degradation by suppressing MMP-13. We also showed that E2 stimulation upregulates the expression levels of miR-140 in chondrocytes. Thus, we speculate that the E2-stimulated increase in miR-140 levels exerts an inhibitory effect on MMP-13.

To further investigate the involvement of miR-140 in the regulation of MMP-13 by E2 in human articular chondrocytes, normal chondrocytes were treated with a miR-140 mimic or a miR-140 inhibitor in the presence of IL-1β, and MMP-13 transcript levels were quantified. Treatment with miR-140 dramatically increased the inhibitory effect of E2 on MMP-13 in IL-1β-treated chondrocytes, whereas treatment with miR-140 inhibitor dramatically enhanced MMP-13 levels with IL-1β treatment. These findings suggest that miR-140 mediates the inhibitory effect of E2 on MMP-13.

Cartilage-specific miR-140 was one of the first miRNAs found to be abundantly expressed in chondrocytes and has been shown to critically regulate cartilage development and homeostasis. Previous studies reported that the proximal region upstream of the pri-miR-140 gene can bind chondrocyte-specific transcriptional factors in vivo. For example, the expression of miR-140 is directly regulated by Sox9 [38, 39]. LSox5 and Sox6 can also control miR-140 expression through a response element in the miR-140 promoter [40].

ER is known to regulate gene transcription by directly binding to the promoter of target genes. The miR-140 promoter sequence contains a classical estrogen response element (ERE) with the consensus sequence 5′-GGTCAnnnTGACC-3′ [41, 42]. Thus, miR-140 could be regulated by binding ER directly. We propose that ER transcriptional activity regulates miR-140 after estradiol-mediated ER activation. Our study demonstrated that miR-140 promoter activity was modulated by estrogen in chondrocytes. Estradiol stimulated miR-140-driven luciferase reporter activity, which suggests that ER can directly activate miR-140 transcription. Therefore, ligand-dependent activation of ER increased ERE-mediated miR-140 promoter activity, resulting in MMP-13 suppression. In menopausal OA conditions, low estrogen levels reduce the induction of ER/miR-140, resulting in increased MMP-13 activity. Thus, this study provides novel insights into how to “fine-tune” ER-dependent signaling for the control of menopausal OA and may facilitate the development of therapeutic approaches based on the modulation of the ER/miR-140 pathway.

**Fig. 6:**
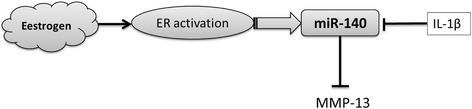
Schematic diagram to show E2 modulation of miR-140 in IL-1β induced MMP-13 expression. *ER* estrogen receptor, *IL-1β* interleukin-1 beta, *MMP-13* metalloproteinase 13

In summary, the present study demonstrates that the ER/miR-140/MMP-13 pathway is responsible for cartilage degradation in human OA chondrocytes. E2 regulates miR-140 and thereby alters MMP-13 activity (Fig. 6). Results from this study suggest that ER/miR-140 may serve as a potential therapeutic target for OA treatment. However, we cannot fully exclude the possibility that other intracellular signaling pathways are also involved in E2-stimulated signal transduction. Our findings also prompted us to investigate whether E2 upregulates miR-140 could attenuates menopausal OA in an OVX mouse model.

## Conclusions

Our data demonstrated that ER regulated the expression of miR-140 in both normal and OA chondrocytes. Furthermore, IL-1β-induced activation of signal transduction pathways associated with the expression of MMP-13 downregulated the expression of miR-140. Thus, ER/miR-140 may play a role in regulating the expression of MMP-13 in human chondrocytes.

## Additional file

Additional file 1: Figure S1. The expression of MMP-13 mRNA level increased in five OA patients’ articular cartilage tissues. Quantitative reverse transcription-polymerase chain reaction analysis of the matrix metalloproteinase 13 (MMP-13) in articular cartilage tissues from five patients with OA. **Figure S2.** The gene expressions of cartilage matrix gene for cartilage development show no effect after E2 treatment. (DOCX 25 kb)

## Abbreviations

3′-UTRs: 3′-untranslated regions
ADAMTS-5: a disintegrin and metalloproteinase with thrombospondin motifs 5
cDNA: complementary DNA
DMEM: Dulbecco’s modified Eagle’s medium
E2: 17-β-estradiol
ECM: extracellular matrix
ER: estrogen receptor
ERE: estrogen response element
FBS: fetal bovine serum
GAPDH: glyceraldehyde 3-phosphate dehydrogenase
IL-1β: interleukin-1 beta
miRNA: microRNA
MMP-13: metalloproteinase 13
mRNA: messenger RNA
OA: osteoarthritis
OVX: ovariectomy
qRT-PCR: quantitative real-time-PCR
siRNA: small interfering RNA
