# Supplementary material for: E2 regulates MMP-13 via targeting miR-140 in IL-1β-induced extracellular matrix degradation in human chondrocytes
Source: Arthritis Res Ther. 2016 May 10;18:105. doi: 10.1186/s13075-016-0997-y (PMC4863330; doi:10.1186/s13075-016-0997-y)
Supplement: Additional file 1: Figure S1. — The expression of MMP-13 mRNA level increased in five OA patients’ articular cartilage tissues. Quantitative reverse transcription-polymerase chain reaction analysis of the matrix metalloproteinase 13 (MMP-13) in articular cartilage tissues from five patients with OA. Figure S2. The gene expressions of cartilage matrix gene for cartilage development show no effect after E2 treatment. (DOCX 25 kb) [file 13075_2016_997_MOESM1_ESM.docx]

Research Highlight

The dramatic rise in OA prevalence among postmenopausal women suggested estrogen deficiency is potential linkage to OA. But the underlying mechanisms of this relationship are unclear. Thus, a better understanding of how estrogen acts on articular chondrocytes in OA is very significant. We reported herein that E2 suppressed MMP-13 transcript expression significantly in articular chondrocytes via increased levels of miR-140.Thus estrogen/ER/miR-140 pathway has been implicated in the process of cartilage degradation in osteoarthritis (OA). This could perhaps open up potential therapeutic strategies such as the development of new specific estrogen ligands as promote miR-140 expression as therapeutic strategies to treat this very prevalent disease.

Figure S1.  The expression of MMP-13 mRNA level increased in five OA patients’ articular cartilage tissues. Quantitative reverse transcription–polymerase chain reaction analysis of the matrix metalloproteinase 13 (MMP-13) in articular cartilage tissues from 5 patients with OA.

Figure S2. The gene expressions of cartilage matrix gene for cartilage development show no affect after E2 treatment.
